# Amyloid Load, Hippocampal Volume Loss, and Diffusion Tensor Imaging Changes in Early Phases of Brain Aging

**DOI:** 10.3389/fnins.2019.01228

**Published:** 2019-11-15

**Authors:** Sven Haller, Marie-Louise Montandon, Cristelle Rodriguez, Valentina Garibotto, Johan Lilja, François R. Herrmann, Panteleimon Giannakopoulos

**Affiliations:** ^1^CIRD Centre d’Imagerie Rive Droite, Geneva, Switzerland; ^2^Department of Surgical Sciences, Radiology, Uppsala University, Uppsala, Sweden; ^3^Faculty of Medicine, University of Geneva, Geneva, Switzerland; ^4^Department of Rehabilitation and Geriatrics, Geneva University Hospitals, University of Geneva, Geneva, Switzerland; ^5^Department of Psychiatry, University of Geneva, Geneva, Switzerland; ^6^Division of Institutional Measures, Medical Direction, Geneva University Hospitals, Geneva, Switzerland; ^7^Division of Nuclear Medicine and Molecular Imaging, Department of Diagnostic, Geneva University Hospitals, Geneva, Switzerland; ^8^Hermes Medical Solutions, Stockholm, Sweden

**Keywords:** amyloid deposition, APOE genotyping, magnetic resonance imaging, normal aging, positron emission tomography

## Abstract

**Background and Purpose:**

Amyloid imaging, gray matter (GM) morphometry and diffusion tensor imaging (DTI) have all been used as predictive biomarkers in dementia. Our objective was to define the imaging profile of healthy elderly controls as a function of their cognitive trajectories and explore whether amyloid burden and white matter (WM) microstructure changes are associated with subtle decrement of neuropsychological performances in old age.

**Materials and Methods:**

We performed a 4.5-year longitudinal study in 133 elderly individuals who underwent cognitive testing at inclusion and follow-up, amyloid PET, MRI including DTI sequences at inclusion, and APOE epsilon 4 genotyping. All cases were assessed using a continuous cognitive score (CCS) taking into account the global evolution of neuropsychological performances. Data processing included region of interest analysis of amyloid PET analysis, GM densities and tract-based spatial statistics (TBSS)-DTI. Regression models were built to explore the association between the CCS and imaging parameters controlling for significant demographic and clinical covariates.

**Results:**

Amyloid uptake was not related to the cognitive outcome. In contrast, GM densities in bilateral hippocampus were associated with worst CCS at follow-up. In addition, radial and axial diffusivities in left hippocampus were negatively associated with CCS. Amyloid load was associated with decreased VBM and increased radial and axial diffusivity in the same area. These associations persisted when adjusting for gender and APOE4 genotype. Importantly, they were absent in amygdala and neocortical areas studied.

**Conclusion:**

The progressive decrement of neuropsychological performances in normal aging is associated with volume loss and WM microstructure changes in hippocampus long before the emergence of clinically overt symptoms. Higher amyloid load in hippocampus is compatible with cognitive preservation in cases with better preservation of GM densities and WM microstructure in this area.

## Introduction

The current use of Alzheimer disease (AD) related amyloid/tau/neurodegeneration profile is made with the *a priori* idea that the first cognitive changes follows the elevation of brain amyloid and accumulation of neurofibrillary tangles (Soldan et al., 2019). In healthy individuals older than 80 years, the concomitant presence of positive biomarkers for amyloid, tau and neurodegeneration is the rule (Jack et al., 2017), still these cases perform within the normal range. However, cognitive trajectories in normal aging are variable ranging from stability over time, initial fluctuations and, in a limited number of cases, progressive worsening of neuropsychological performances that may take place long before the transition to the mild cognitive impairment (MCI) state. Although crucial for our understanding of the initial stages of neurodegeneration, the neuroimaging correlates of the individual trajectories within the normal range are poorly explored. The variability of clinical definitions before the appearance of the first cognitive deficits as well as limited follow-up period in this long asymptomatic phase are two major methodological problems facing this endeavor.

Among the different imaging technics that could be used to predict the cognitive trajectories in normal aging, amyloid positron emission tomography (PET) imaging, magnetic resonance imaging (MRI), and diffusion tensor imaging (DTI) assessing white matter (WM) and gray matter (GM) microstructure are likely to address at least partly temporally distinct processes (Bosch et al., 2012). Based on 1209 cognitively intact individuals aged 50–95, Jack et al. (2015) reported that hippocampal volume loss might occur before abnormal amyloid PET occurrence. Unlike hippocampal volume decrease that usually becomes significant after 60 years of age and is APOE4-independent, amyloid PET positivity is more frequent after 70 years of age with an increase of amyloid burden in the presence of APOE4 allele. These data indicated that Aß accumulation arises later in life on a background of preexisting structural deficits that are associated with normal aging and not with amyloid pathology *per se* (Jack et al., 2015). Early studies exploring the relationship between WM and amyloid have combined DTI and *ex vivo* histopathology. A positive relationship between the presence of amyloid deposits and WM microstructure abnormalities has been reported (Song et al., 2004; Sun et al., 2005; Shu et al., 2013; Zerbi et al., 2013; Racine et al., 2014; Wolf et al., 2015; Vipin et al., 2019). Importantly, the imaging parameters cited above are partly interdependent. The presence of hippocampal atrophy has been shown to impact on the association between DTI findings and amyloid positivity (Kantarci et al., 2014). Low amyloid burden is associated with higher FA values as a possible compensatory phenomenon at early stages of brain aging (Wolf et al., 2015).

Although within normal age-adjusted performances, elderly individuals with slowly declining cognitive abilities may exhibit a distinct pattern of amyloid deposition, volumetric and WM microstructure changes (Downer et al., 2017; Lin et al., 2017; Min et al., 2018). The present longitudinal study of a community-based cohort of 133 highly educated elderly controls focuses on their cognitive trajectories during a 4.5 year follow-up and explores their association with patterns of amyloid deposition, GM densities, and DTI markers and in this context. All of the cases were assessed with PET amyloid scans (for early amyloid burden), tract-based spatial statistics DTI (for WM microstructure), and GM densities (for brain atrophy) at inclusion and were classified according to their neuropsychological performances over the follow-up period.

Our main hypothesis is that cognitive fluctuations within the normal range are not innocuous and may be associated with significant GM and WM damage in key cortical areas for AD pathogenesis. We also hypothesized that amyloid load did not affect cognition in this highly selected series of healthy elders.

## Materials and Methods

### Participants

The study was approved by the local Ethics Committee and all participants gave written informed consent prior to inclusion. Individuals were selected from an ongoing cohort study on cognitively intact elders, as described in detail previously (Xekardaki et al., 2015; Zanchi et al., 2017; van der Thiel et al., 2018). Cases with three neurocognitive assessments at baseline, 18 months and 54 months, brain amyloid PET, structural brain MRI and DTI at inclusion and APOE status were considered. Exclusion criteria included psychiatric or neurologic disorders, sustained head injury, history of major medical disorders (neoplasm or cardiac illness), alcohol or drug abuse, regular use of neuroleptics, antidepressants or psychostimulants and contraindications to PET or MR imaging. To control for the confounding effect of vascular pathology on DTI findings, individuals with subtle cardiovascular symptoms, hypertension (non-treated), and a history of stroke or transient ischemic episodes were also excluded from the present study. The final sample included 133 elderly individuals (mean age 76.8 ± 4.0 years, 84 females).

### Neuropsychological Assessment

At baseline, all individuals were evaluated with an extensive neuropsychological battery, including the mini-mental state examination (MMSE) (Folstein et al., 1975), the Hospital Anxiety and Depression Scale [HAD (Zigmond and Snaith, 1983], and the Lawton instrumental activities of daily living [IADL (Barberger-Gateau et al., 1992)]. Cognitive assessment included (a) attention (Digit-Symbol-Coding (Wechsler, 1997), Trail Making Test A (Reitan, 1958), (b) working memory [verbal: Digit Span Forward (Wechsler, 1955)], visuo-spatial: visual memory span (Corsi) (Milner, 1971), (c) episodic memory [verbal: RI-48 Cued Recall Test (Buschke et al., 1997)], visual: Shapes Test (Baddley et al., 1994), (d) executive functions [Trail Making Test B (Reitan, 1958), Wisconsin Card Sorting Test and Phonemic Verbal Fluency Test (Heaton, 1981)], (e) language [Boston Naming (Kaplan et al., 1983)], (f) visual gnosis (Ghent Overlapping Figures), (g) praxis: ideomotor (Schnider et al., 1997), reflexive (Poeck, 1985), and constructional [Consortium to Establish a Registry for Alzheimer’s Disease (CERAD), Figures copy (Welsh et al., 1994)]. All individuals were also evaluated with the Clinical Dementia Rating scale (CDR) (Hughes et al., 1982). In agreement with the criteria of Petersen et al. (Petersen et al., 2001), participants with a CDR of 0.5 but no dementia and a score exceeding 1.5 standard deviations below the age-appropriate mean in any of the cognitive tests were classified as MCI and were excluded. Participants with neither dementia nor MCI were classified as cognitively healthy controls and underwent full neuropsychological assessment at follow-ups, on average 18 and 54 months later.

The subtle cognitive decline was defined according to a continuous cognitive score (CCS), computed as follows. Most of the cognitive performances, discrete or continuous, cannot be linearly combined by adding the individual scores to a unique composite cognitive score. Thus, all values were converted to *z* scores. Subsequently, we summed the number of cognitive tests at follow-up with performances at least 0.5 standard deviation (SD) higher compared with the first evaluation, leading to the number of tests with improved performances (range, 0–14). Similarly, we summed the number of cognitive tests at follow-up with performances at least 0.5 SDs lower compared with the first evaluation, yielding the number of tests with decreased performances (range 0–14). Finally, the number of tests with improved minus the number of tests with decreased performances results in a final CCS. Change in cognition between inclusion and last follow-up was defined as the sum of the CCSs at two follow-ups.

### Amyloid PET Imaging

Seventy-seven ^18^F-Florbetapir- (Amyvid) and fifty-six ^18^F-Flutemetanol-PET (Vizamyl) data were acquired on 2 different instruments (Siemens BiographTM mCT scanner and GE Healthcare Discovery PET/CT 710 scanner) of varying resolution and following different platform-specific acquisition protocols. The ^18^F-Florbetapir images were acquired 50–70 min after injection and the ^18^F-Flutemetanol images 90–120 min after injection. PET images were reconstructed using the parameters recommended by the ADNI protocol aimed at increasing data uniformity across the multicenter acquisitions. More information on the different imaging protocols for PET acquisition can be found on the ADNI web site^1^.

### MR Imaging

At baseline, imaging data were acquired on a 3T MRI scanner (TRIO SIEMENS Medical Systems, Erlangen, Germany). The structural high-resolution T1-weighted anatomical scan was performed with the following fundamental parameters: 256 × 256 matrix, 176 slices, 1 mm isotropic, TR = 2.27 ms). An additional DTI sequence was acquired (*b* = 0 and 30 diffusion directions with *b* = 1000 s/mm^2^, 128 × 128 matrix, voxel size 2.0 mm^3^ × 2.0 mm^3^ × 2.0 mm^3^, TE = 74.5 ms, TR = 15809 ms and 1 average). Additional sequences included axial fast spin-echo T2w imaging and susceptibility weighted imaging to exclude brain disease, such as ischemic stroke, subdural hematomas, or space-occupying lesions.

### APOE Assessment

Whole blood samples were collected at baseline for all subjects for APOE genotyping. Standard DNA extraction was performed using either 9 ml EDTA tubes (Sarstedt, Germany) or Oragene Saliva DNA Kit (DNA Genotek, Inc., Ottawa, ON, Canada) which were stored at −20°C. APOE genotyping was done on the LightCycler (Roche Diagnostics, Basel, Switzerland) as described previously (Nauck et al., 2000). Subjects were classified according to the presence of an APOEε4 allele (ε4/ε3, ε3/ε3, and ε3/ε2 carrier).

### Amyloid PET Preprocessing and Analysis

All scans were intensity normalized using a modified Centiloid pons as reference region created by Lilja et al. (2018). The voxel-wise analysis was carried out using the FSL software package^2^, and the voxel-wise FSL General Linear Model was applied by using permutation-based non-parametric testing with the FSL Randomize Tool with the threshold-free cluster enhancement (TFCE) correction for multiple comparisons (Smith and Nichols, 2009) considering fully corrected *p* values <0.05 as significant. The analysis was performed across all participants across the entire brain using the CCS as explanatory variable, and age, gender, education, APOE status and tracer as non-explanatory variables.

### Preprocessing of DTI Data

Preprocessing of the DTI data was performed by using the standard procedure of TBSS, as described in detail before (Smith et al., 2006), in the FSL software package^2^. All subjects’ fractional anisotropy (FA) data were projected onto a mean FA skeleton using a non-linear spatial registration. The FA represents an index for the amount of diffusion asymmetry within a voxel. The tract skeleton is the basis for voxel-wise cross-subject statistics and reduces potential misregistrations as the source for false-positive or false-negative analysis results. The other DTI-derived parameters, axial diffusivity (the first eigenvalue, AR) and radial diffusivity (the average of the second and third eigenvalues, RD), were analyzed in the same way by using spatial transformation parameters that were estimated in the initial FA analysis.

### Region of Interest (ROI) Analysis

We performed a ROI analysis on amyloid load and GM densities in 9 regions of particular interest in the context of cognitive decline (posterior cingulate cortex, mesial temporal lobe, parietal lobe, as well as hippocampus, amygdala, and caudate nucleus bilaterally) with the occipital lobe as control region. The ROI analysis was also performed on FA, AD, and RD values in all of the above mentioned areas.

### Statistical Analysis

Male and female participants’ characteristics were compared using Fisher exact, Mann. Whitney *U* or *t* tests as appropriate. The significance level was set at *P* < 0.05. Simple and multiple linear regression models were used to predict CCS (dependent variable) with amyloid load, GM densities and DTI markers (FA, AD, and RD) after including APOE epsilon 4 status (presence/absence), and gender as independent predictors. Only covariates that were significantly associated with the CCS in univariate models were included in multivariate models. A threshold of P value less than 0.05 was applied for significance. The correlation between amyloid values and TBSS-DTI, GM densities was assessed using Spearman rank correlation coefficient. The “Stata” software release 16.0 was used for all analyses.

## Results

### Demographic Data

The main demographic data is summarized in Table 1. Importantly, no case evolved to MCI during the follow-up period. Men were significantly more educated than women (*p* = 0.0013). The CCS (combined on the basis of two follow-ups) decreased in men but remained fairly stable in women (*p* = 0.038). The mean SUVr in bilateral hippocampus was below 0.8 in all of the cases implying a low level of amyloid deposition in these cognitively preserved cases.

**TABLE 1:** Gender-related differences in demographic and clinical data, radiological parameters, and APOE status.

	**Gender**			
	**Female**	**Male**	**Total**	***p* Value**
*N*	84	49	133	
Age at amy PET	77.8 ± 4.4	78.0 ± 3.8	77.9 ± 4.2	0.7469
Age at MRI	76.8 ± 4.2	76.9 ± 3.8	76.8 ± 4.0	0.8421
CCS	−0.1 ± 3.4	−1.4 ± 3.6	−0.6 ± 3.5	0.0375
Education (year)				0.0013
<9	17 (20.2%)	1 (2.0%)	18 (13.5%)	
9–12	41 (48.8%)	22 (44.9%)	63 (47.4%)	
>12	26 (31.0%)	26 (53.1%)	52 (39.1%)	
Fazekas at 1st MRI				0.6577
0	22 (38.6%)	15 (40.5%)	37 (39.4%)	
1	21 (36.8%)	15 (40.5%)	36 (38.3%)	
2	10 (17.5%)	6 (16.2%)	16 (17.0%)	
3	4 (7.0%)	1 (2.7%)	5 (5.3%)	
Mean hippocampal SUVr				
Left hippocampus	0.61 ± 0.07	0.60 ± 0.06	0.60 ± 0.06	0.2403
Right hippocampus	0.62 ± 0.07	0.60 ± 0.06	0.61 ± 0.07	0.1750
APOE				0.3305
Negative	53 (82.8%)	37 (88.1%)	90 (84.9%)	
Positive	11 (17.2%)	5 (11.9%)	16 (15.1%)	

### Amyloid Deposition, GM Densities, and DTI Markers as a Function of the CCS Score

**FIGURE 1:**
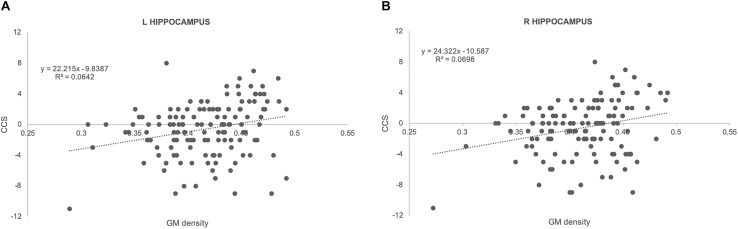
Scatterplots illustrating the association between GM densities and CCS together with best fit equations for left **(A)** and right **(B)** hippocampus.

**FIGURE 2:**
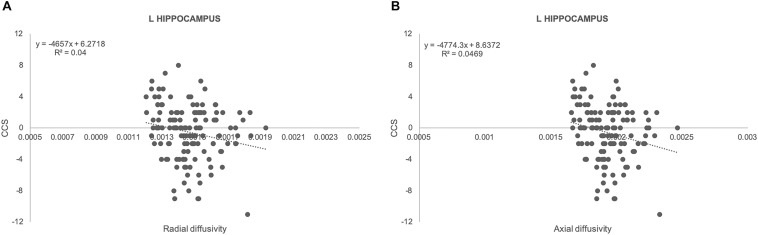
Scatterplots illustrating the association between radial **(A)** and axial **(B)** diffusivity and CCS together with best fit equations for left hippocampus.

There was no significant association between amyloid load and CCS in all of the areas studied. In contrast, there were significant positive associations between ROI measures of GM densities in hippocampus bilaterally and CCS Figure 1; left: regression coefficient: 22.2 (CI: 7.55, 36.87), *p* = 0.003; right: regression coefficient: 24.32 (CI: 8.98, 39.67), *p* = 0.002]. Results from TBSS-DTI analysis showed significant negative associations between both radial [regression coefficient: −4657 (CI: −8598, −715), *p* = 0.021] and axial diffusivity [regression coefficient: −4774 (CI: −8494, −1053), *p* = 0.012] in left hippocampus and CCS scores (Figure 2). FA analysis did not reveal significant associations with the cognitive outcome. Importantly, these associations persisted after adjustment for gender (the only demographic variable that was associated with imaging parameters in this sample) and APOE 4 genotype.

### Correlation Between Amyloid Tracer Uptake and GM Densities, DTI Markers

**FIGURE 3:**
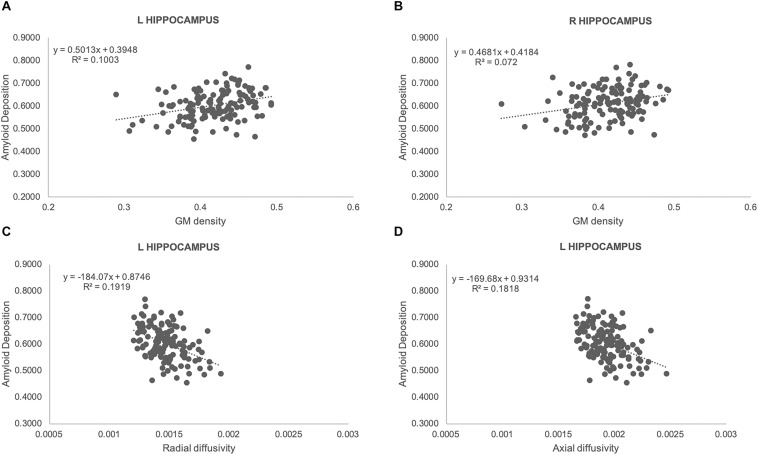
Scatterplots illustrating the association between amyloid load and GM densities **(A,B)**, radial **(C)** as well as axial **(D)** diffusivities for left **(A,C,D),** and right **(B)** hippocampus. Best fit equations are integrated in each plot.

We observed a significant positive correlation between GM densities and amyloid load in both left (*r*_*s*_: 0.32, *p* = 0.0002) and right (*r*_*s*_: 0.27, *p* = 0.0018) hippocampus (Figures 3A,B). Moreover, a negative association was found between amyloid values and both radial (*r*_*s*_: −0.44, *p* = 0.0001) and axial diffusivity (*r*_*s*_: −0.43, *p* = 0.0001) in left hippocampus (Figures 3C,D).

## Discussion

Our findings reveal that the subtle changes in cognitive performances that occur during a long follow-up in normal aging are associated with decreased GM densities and altered WM microstructure in hippocampus. They also indicate that the low amyloid load observed in our community-based cohort of healthy elders has no direct impact on cognition and did not affect GM and WM integrity. In contrast to the majority of previous investigations, we did not include AD or MCI cases, but focused solely on cognitively preserved elderly individuals without significant vascular burden that remained within the normal range during the study period. Even the cases with the more marked decrement of their CCS with respect to their own baseline evaluation did not satisfy the MCI criteria even 4.5 years post-inclusion. Subsequently, our observations concern the initial stages of brain aging long before the emergence of clinically overt cognitive dysfunction. In this critical period, cognitive trajectories are still not fully determined by the impact of neurodegenerative changes and compensation abilities may be sufficiently active to counterbalance the deleterious effect of lesion accumulation.

Current evidences about the role of amyloid accumulation in these individuals remain ambiguous. Although several data suggested that elevated amyloid levels at baseline (SUVr > 1.5) was associated with greater cognitive decline at follow-up (Petersen et al., 2016), the INSIGHT-pre-AD data published recently showed no association between this parameter and cognitive fate at 30-month follow-up in healthy controls (Dubois et al., 2018). Another recent contribution indicated that PIB PET β-amyloid’s relationship to cognitive decline was non-linear being more prominent at lower β-amyloid levels (Knopman et al., 2018). Consistent with the INSIGHT-pre-AD data, amyloid load was not related to the cognitive outcome in the present series. One possible explanation for this finding may reside to the low levels of mean SUVr in our cases (less than 0.8 in hippocampus). It is thus likely that such small amyloid uptake is not sufficient for a direct deleterious effect on cognitive performances.

With respect to MRI data, there was a significant association between GM densities in both hippocampus and cognitive status in the present series. Data on GM evolution in normal aging remains ambiguous. Regional GM decrements in right thalamus, left parahippocampal gyrus, inferior temporo-parietal lobules, anterior cingulum, and precentral gyrus have been documented (Lee et al., 2016; Fletcher et al., 2018; Squarzoni et al., 2018) but in certain cohorts GM densities were preserved or marginally affected independently of the cognitive performances of healthy controls (Hong et al., 2016; Takeuchi et al., 2017). Unlike FA, the increase of AD and RD in left hippocampus reflects the presence of early axonal and myelin damage in asymptomatic cases with progressive decrement of cognitive performances prior to the MCI status. This finding supports the idea that early changes of diffusivity parameters in hippocampus may be more efficient than FA as a surrogate marker of cognitive dysfunction in non-demented individuals (Fellgiebel and Yakushev, 2011).

Whether amyloid load impacts on hippocampal volumes in cognitively preserved individuals is still matter of debate. Previous studies implied that this could be the case only in some vulnerable individuals (Nosheny et al., 2015; Khan et al., 2017; Duarte-Abritta et al., 2018). Equally ambiguous is the association between DTI parameters and amyloid load in normal aging. Some authors pointed to a RD increase with high amyloid uptake (Wolf et al., 2015) whereas others found compensatory increase of FA associated with impaired diffusion in amyloid positive cases (Racine et al., 2014). Negative data were also reported supporting an independent effect of DTI changes and amyloid deposits on cognitive decline in preclinical AD cases (Kantarci et al., 2014). Our correlation analysis showed that PET amyloid uptake was negatively related to both AD and RD in left hippocampus and positively associated with GM densities in hippocampus bilaterally. These results imply that higher amyloid load in hippocampus is compatible with cognitive preservation in cases with better preservation of GM densities and WM microstructure in this area. The absence of a direct toxic effect of amyloid on WM microstructure and GM densities supports the dissociation between neurodegeneration, WM microstructure changes and amyloid pathology in the initial phases of brain aging (Insel et al., 2015). In the same line, Wang et al., showed that elevated CSF tau in healthy elders is associated with cortical thinning but not the classical hippocampal atrophy. An inverse pattern was observed in cases with decreased CSF Aß42 implying the presence of a spatially distinct neurodegeneration related to Aß and tau pathology in preclinical AD (Wang et al., 2015). Analyzing 570 ADNI controls, Edmonds et al. (2015) reported that among the cases with only one abnormal biomarker at baseline that later progress to MCI, neurodegeneration alone was much more frequent than amyloidosis or subtle cognitive deficits which were equally common. Besson et al. (2015) postulated that most preclinical AD cases have either neurodegeneration or Aß deposition but not both. These observations point to the necessity to add imaging modalities such as tau imaging and fluorodeoxyglucose PET in order to assess the complex relationships between GM densities/DTI parameters and the development of AD pathology in normal brain aging.

Strengths of the present study includes the combination of three imaging modalities in the initial stages of normal brain aging (amyloid PET, GM morphometry, DTI), longitudinal assessment of neuropsychological performances using a detailed battery, and exclusion of cases with cardiovascular risk factors or significant vascular pathologies that could affect DTI data. Two limitations should, however, be taken into account. We report here the results of a cohort of highly educated elders without significant vascular burden that is clearly not representative of the whole spectrum of normal aging. The paucity of DTI alterations and in particular FA decrease, contrasts with previous reports in brain aging (for review see Squarzoni et al., 2018) and may reflect a selection bias and should thus be interpreted with caution. Finally, although our follow-up lasted for 4.5 years, we cannot exclude that early amyloid burden impacts on DTI-assessed longitudinal WM changes at later time points (Vipin et al., 2019).

## Conclusion

Our data shed new light into the complex association between neuroimaging markers and cognitive trajectories in normal brain aging. Although not yet considered pathological, the continuous decrement of cognitive performances in this community-based cohort is associated with substantial changes in morphometry and WM diffusivity parameters in hippocampus. The low amyloid load in these cases did not affect cognition. Its association with higher GM densities and lower RD and AD values in hippocampus support the dissociation between amyloid pathology and neurodegeneration in normal brain aging.

## Data Availability Statement

The raw data supporting the conclusions of this manuscript will be made available by the authors, without undue reservation, to any qualified researcher.

## Ethics Statement

The studies involving human participants were reviewed and approved by the Commission Cantonale d’Ethique de la Recherche (CCER) Geneva. The patients/participants provided their written informed consent to participate in this study.

## Author Contributions

SH, M-LM, JL, and FH performed and analyzed the data. SH, PG, and M-LM wrote the manuscript. CR and VG were involved in participants recruitment, follow-up, and data acquisition. All authors read and approved the final manuscript.

## Conflict of Interest

The authors declare that the research was conducted in the absence of any commercial or financial relationships that could be construed as a potential conflict of interest.

## Footnotes

1 http://adni.loni.usc.edu/methods/

2 http://www.fmrib.ox.ac.uk/fsl/
